# Invasive pneumococcal infections among persons with and without underlying medical conditions: Implications for prevention strategies

**DOI:** 10.1186/1471-2334-8-96

**Published:** 2008-07-22

**Authors:** Peter Klemets, Outi Lyytikäinen, Petri Ruutu, Jukka Ollgren, J Pekka Nuorti

**Affiliations:** 1 National Public Health Institute (KTL), Department of Infectious Disease Epidemiology and Control, Helsinki, Finland; 2 Respiratory Diseases Branch, National Center for Immunization and Respiratory Diseases, Centers for Disease Control and Prevention (CDC), Atlanta, GA, USA

## Abstract

**Background:**

The 23-valent pneumococcal polysaccharide vaccine (PPV23) is recommended for persons aged < 65 years with chronic medical conditions. We evaluated the risk and mortality from invasive pneumococcal disease (IPD) among persons with and without the underlying medical conditions which are considered PPV23 indications.

**Methods:**

Population-based data on all episodes of IPD (positive blood or cerebrospinal fluid culture) reported by Finnish clinical microbiology laboratories during 1995–2002 were linked to data in national health care registries and vital statistics to obtain information on the patient's preceding hospitalisations, co-morbidities, and outcome of illness.

**Results:**

Overall, 4357 first episodes of IPD were identified in all age groups (average annual incidence, 10.6/100,000). Patients aged 18–49 and 50–64 years accounted for 1282 (29%) and 934 (21%) of IPD cases, of which 372 (29%) and 427 (46%) had a current PPV23 indication, respectively. Overall, 536 (12%) IPD patients died within one month of first positive culture. Persons aged 18–64 years accounted for 254 (47%) of all deaths (case-fatality proportion, 12%). Of those who died 117 (46%) did not have a vaccine indication. In a survival model, patients with alcohol-related diseases, non-haematological malignancies, and those aged 50–64 years were most likely to die.

**Conclusion:**

In the general population of non-elderly adults, almost two-thirds of IPD and half of fatal cases occurred in persons without a recognised PPV23 indication. Policymakers should consider additional prevention strategies such as lowering the age of universal PPV23 vaccination and introducing routine childhood pneumococcal conjugate immunisation which could provide substantial health benefits to this population through indirect vaccine effects.

## Background

*Streptococcus pneumoniae *is a leading cause of serious community-acquired infections such as bacteraemia and meningitis and community-acquired pneumonia. The pneumococcal polysaccharide vaccine (PPV23) is recommended for adults aged 18–64 years with certain chronic medical conditions and elderly persons because of high disease rates and increased risk of death [1,2]. Available information from representative, population-based studies on the outcome of IPD is limited. Case fatality has generally been defined as in-hospital mortality, without extended follow up after discharge from hospital [1,2].

The estimates of PPV23 effectiveness against IPD are highest among otherwise healthy young adults [3-6], but lower among persons with multiple underlying medical conditions and immunosuppression [7,8]. In the United States, routine childhood 7-valent pneumococcal conjugate vaccine (PCV7) immunisation program has resulted in dramatic reductions in rates of pneumococcal-related diseases and major changes in the epidemiology of pneumococcal infections in children and adults because of reduced transmission of vaccine types and herd protection in unvaccinated groups [9-11]. In Finland, however, PCV7 has not been introduced in the national immunisation schedule and the uptake of PPV23 in the recommended target groups has been extremely low (3%) [12].

We investigated all first episodes of IPD identified by Finnish microbiology laboratories during 1995–2002 and linked these national, population-based surveillance data to other health registries to evaluate the risk and outcome of IPD among patients with various underlying medical conditions with special emphasis on the general population of working-age adults.

## Methods

### Surveillance

The Finnish National Health Care System is organised into 20 geographically and administratively defined health care districts (HCD), with catchment populations ranging from 68,000 to 1.4 million (total population 5.2 million). All clinical microbiology laboratories are required to notify bacterial isolations from blood and cerebrospinal fluid (CSF), including *S. pneumoniae*, to the National Infectious Disease Register (NIDR), primarily through electronic reporting. Each notification includes information on the date and type of specimen, date of birth, sex, and place of treatment. Using this information and a time interval of three months, possible multiple positive culture results or notifications concerning the same individual are merged into a single case either by a notifying laboratory or after the notification is received in the NIDR database.

### Study population and definitions

A case with IPD was defined as isolation of *S. pneumoniae *from blood and/or CSF during 1995–2002. Of the total of 4611 IPD episodes identified by the primary diagnostic laboratory, only the first episode from each case-patient was included in the analysis (N = 4357); recurrent episodes and those with missing identification information were excluded. Pneumococcal bacteraemia was defined as isolation of *S. pneumoniae *from blood only and pneumococcal meningitis as isolation of *S. pneumoniae *from CSF with or without pneumococcal bacteraemia within 7 days. Patients with a vaccine indication were defined as those who had at least one of the underlying medical conditions for which PPV23 is recommended in Finland (Additional file 1). This list of conditions is practically identical to that recommended by the U.S. Advisory Committee on Immunization Practices (ACIP) [13].

### Underlying medical conditions

Information on co-morbidities and underlying conditions for IPD patients was obtained by linking the IPD surveillance database to the following national population-based registries using the date of the first positive specimen for *S. pneumoniae *and the national identity code: the Cancer Registry (diagnosis of haematological and non-haematological malignancy within one year and five years prior to the specimen date), National Social Insurance Institution (KELA), National Hospital Discharge Register (HILMO) and NIDR (HIV infection). Presence of diabetes mellitus, chronic pulmonary disease (chronic obstructive pulmonary disease [COPD] and asthma), congenital or acquired immunodeficiency, rheumatic or other autoimmune diseases requiring immunosuppressive therapy, solid organ or bone marrow transplantation, cardiac failure, or renal failure were defined as a KELA record indicating entitlement for reimbursement of medications for these conditions. Alcohol-related diseases (ARD), chronic liver diseases, diseases of the spleen, and CSF leak were defined as records in HILMO with one ore more International Classification of Diseases (ICD), Ninth or 10^th ^Revision (from 1996 onward) coded discharge diagnoses within one year before the first positive specimen date (ICD-codes in Additional file 2).

Vital status (possible date of death) at 7, 28 and 90 days from the first positive culture of *S. pneumoniae *for each case-patient was determined from the National Population Information System.

### Calculation of incidence rates and statistical analysis

Annual population data from the Statistics Finland during 1995–2002 were used as denominators to calculate age- and sex-specific incidence rates. In the same way, the denominators for population at risk in person years for categories of underlying medical conditions were obtained from corresponding national health care registries. Categorical variables were analyzed with the χ^2 ^test or Fisher's exact test. Continuous variables were analyzed by the Mann-Whitney *U *test. *P *< .05 was considered to be statistically significant. Piecewise exponential hazard regression model [14] was used in age-group 18–64 years to assess the risk of death with underlying medical conditions for which PPV23 is currently recommended in Finland (Additional file 1), controlling also for other medical underlying conditions, the type of IPD presentation, age and sex. Data were analysed by using SPSS for Windows version 14.0 (Chicago, IL, USA) and Stata 8.2 (College Station, TX, USA).

### Ethical aspects

Use of data collected from population-based registries in this research was authorised by the Ministry of Social Affairs and Health, the Finnish Data Protection Authority, and the National Research and Development Center for Welfare and Health.

## Results

### Incidence

During 1995–2002, 4357 first episodes of IPD were identified in all age groups and 2216 (51%) were persons aged 18–64 years; 1282 (29%) and 934 (21%) were aged 18–49 years and 50–64 years, respectively. The average annual incidence of IPD was 10.6 cases per 100,000 persons. The median age of cases was 53 years (range, 0–98 years) and 2536 (58%) were males. The median duration of hospitalisation was 7 days (range, 0–530 days).

Of the cases in all age groups, 4106 (94%) were bacteraemias and 251 (6%) meningitis. Within the age group 18–64 years, the rates of bacteraemia and meningitis increased 2.5 and 3.3 fold with increasing age (Table 1). The rate of bacteraemia was significantly higher in males than in females in all age groups; for meningitis the rate was higher only in males aged 35–49 years.

**Table 1:** Incidence of *Streptococcus pneumoniae *bacteraemia and meningitis by age and sex, Finland, 1995–2002

	Rate^a ^of bacteraemia (no of cases)					Rate^a ^of meningitis (no of cases)				
		
Age group (years)	Male	Female	Total	Rate ratio^b^	95% CI^c^	Male	Female	Total	Rate ratio^b^	95% CI^c^
0–17	8.3 (386)	6.0 (299)	7.2 (685)	1.4	1.2–1.6	0.5 (22)	0.6 (26)	0.5 (48)	0.8	0.5–1.4
18–34	5.7 (265)	3.2 (142)	4.5 (407)	1.8	1.5–2.2	0.4 (17)	0.1 (6)	0.3 (23)	4.0	0.7–10.1
35–49	11.3 (544)	5.3 (245)	8.4 (789)	2.1	1.8–2.5	0.9 (43)	0.4 (20)	0.7 (63)	2.2	1.3–3.8
50–64	14.6 (546)	8.2 (314)	11.4 (860)	1.8	1.5–2.0	1.1 (42)	0.8 (32)	1.0 (74)	1.4	0.9–2.2
65–74	23.0 (349)	13.9 (275)	17.9 (624)	1.7	1.4–1.9	0.7 (10)	0.9 (17)	0.8 (27)	0.8	0.4–1.7
≥ 75	37.4 (304)	24.1 (437)	28.2 (741)	1.6	1.3–1.8	1.0 (8)	0.4 (8)	0.6 (16)	2.5	0.9–6.7
All	11.9 (2394)	8.1 (1712)	9.9 (4106)	1.5	1.4–1.6	0.7 (142)	0.5 (109)	0.6 (251)	1.4	1.1–1.8

^a^Average annual incidence (cases per 100,000 population); ^b^male to female rate ratio; ^c^CI, confidence interval

### Underlying conditions

In all age-groups, 2302 (53%) of cases had at least one underlying condition, including age ≥ 65 years, for which PPV23 is recommended. However, among working-age patients aged 18–64 years, only 799 (36%) had a PPV23 indication. Of patients aged 18–49 and 50–64 years 372 (29%) and 427 (46%), respectively, had a condition for which PPV23 is currently recommended. Among working-aged persons, alcohol-related diseases, chronic pulmonary disease and diabetes mellitus were the most common vaccine indications whereas cardiac failure, chronic pulmonary disease and diabetes mellitus were most common among elderly patients (Table 2). Persons aged 16–84 years accounted for 96% of cases with alcohol-related diseases. The highest rates of IPD were seen in patients with haematological malignancy and organ or bone marrow transplantation.

**Table 2:** Risk of invasive *Streptococcus pneumoniae *infection (IPI) associated with selected underlying conditions, Finland, 1995–2002.

Underlying condition^a^	Number of IPI cases by age group (years)				Population at risk (person years)	Rate/100,000/year	95%CI^b^	Case fatality proportion at day 7/28/90
					
	< 18	18–64	≥ 65	All (%)				
					
	N = 733	N = 2216	N = 1408	N = 4357				
Chronic pulmonary disease^c^	44	190	260	494 (11.3)	1,435,000	34.4	31.4–37.4	8/12/15
Cardiac failure	13	51	287	351 (8.1)	746,000	47.1	42.2–52.0	16/24/30
Diabetes mellitus	3	140	186	329 (7.5)	1,014,000	32.5	29.0–36.0	13/19/23
Type 1 diabetes^d^	3	16	0	19 (0.4)	158,000	12.0	6.6–17.4	5/5/5
Immunodeficiency or rheumatic diseases	4	128	138	270 (6.2)	779,000	34.7	30.5–38.9	14/19/22
Alcohol-related diseases	0	241	10	251 (5.8)	1,117,000^e^	21.9^f^	19.1–24.6	21/28/33
Non-haematological malignancy								
< 1 year since diagnosis	2	46	58	106 (2.4)	208,000	50.9	41.2–60.6	22/29/41
< 5 years since diagnosis	13	92	139	244 (5.6)	730,000	33.4	29.2–37.6	16/24/33
Haematological malignancy								
< 1 year since diagnosis	5	25	26	56 (1.3)	10,000	547.2	398.9–685.5	14/16/25
< 5 years since diagnosis	23	68	72	163 (3.7)	38,000	434.5	367.8–501.2	10/13/21
Organ or bone marrow transplantation	5	26	3	34 (0.8)	21,000	163.7	108.7–218.7	3/6/9
Chronic renal failure	1	15	8	24 (0.6)	27,000	88.6	53.1–124.1	8/13/17
Chronic liver disease	0	15	5	20 (0.5)	NA^g^	NA	NA	15/30/35
HIV infection	0	10	0	10 (0.2)	8,000	129.7	49.3–210.1	10/20/30

^a^Patient may have more than one underlying condition; ^b^CI, confidence interval; ^c^chronic obstructive pulmonary disease (COPD) and/or bronchial asthma; ^d^diabetes mellitus diagnosed at age < 30 years and insulin treatment; ^e^extrapolation based on 12 month-prevalence of persons with alcohol use disorders from a representative sample of Finland's adult (≥ 30 years) population [21]; ^f^IPI cases with alcohol-related diseases aged ≥ 30 years (N = 245); ^g^NA, not available

### Mortality

Of the 4357 cases with IPD, 202 (5%) died on the day of admission, 373 (9%) died within one week, and 536 (12%) within 28 days. An additional 130 cases died between 29–90 days after the first blood- and/or CSF-culture positive for *S. pneumoniae *(Table 3). Four hundred and thirty-seven (10%) cases died during the hospitalisation for IPD. The in-hospital case-fatality proportions (CFP) within 7, 28 and 90 days were 8%, 10% and 11%, respectively.

**Table 3:** Outcome of *Streptococcus pneumoniae *bacteraemia and meningitis by age and sex, Finland, 1995–2002.

	Number of deaths (case fatality proportion, %)											
	
	Bacteremia						Meningitis					
		
	7 days		28 days		90 days		7 days		28 days		90 days	
						
Age group (years)	Male	Female	Male	Female	Male	Female	Male	Female	Male	Female	Male	Female
0–17	2 (0.6)	4 (1.3)	2 (0.6)	4 (1.3)	2 (0.6)	4 (1.3)	1 (4.5)	2 (7.7)	1 (4.5)	2 (7.7)	1 (4.5)	2 (7.7)
18–34	9 (3.4)	6 (4.2)	15 (5.7)	7 (4.9)	15 (5.7)	8 (5.6)	0 (0.0)	1 (16.7)	2 (11.8)	2 (33.3)	2 (11.8)	2 (33.3)
35–49	43 (7.9)	22 (8.9)	59 (10.9)	27 (11.0)	67 (12.3)	33 (13.5)	4 (9.3)	3 (15.0)	8 (18.6)	4 (20.0)	9 20.9)	5 (25.0)
50–64	53 (9.7)	21 (6.7)	92 (16.7)	28 (8.9)	114 (20.9)	39 (12.4)	4 (9.5)	0 (0.0)	7 (16.7)	3 (9.4)	12 (28.6)	4 (12.5)
65–74	39 (11.2)	26 (9.4)	55 (15.8)	37 (13.4)	76 (21.8)	47 (17.1)	2 (20.0)	4 (23.5)	3 (30.0)	5 (29.4)	3 (30.0)	5 (29.4)
≥ 75	65 (21.4)	58 (13.3)	84 (27.6)	83 (19.0)	100 (32.9)	109 (24.9)	2 (25.0)	2 (25.0)	3 (37.5)	3 (37.5)	3 (37.5)	4 (50.0)
All	211 (8.8)	137 (8.0)	307 (12.8)	186 (10.9)	374 (15.6)	240 (14.0)	13 (9.1)	12 (11.0)	24 (16.9)	19 (17.4)	30 (21.1)	22 (20.2)

Of all deaths during the first month, persons aged 18–49 and 50–64 years accounted for 124 (23%) and 130 (24%); CFP were 10% and 14%, respectively. The CFP at 7, 28 and 90 days were similar for both sexes in the age group 18–49 years but significantly higher in men than in women in persons aged 50–64 years at 28 and 90 days (17% vs. 9% and 21% vs. 12%; p < 0.01 for both comparisons). The CFP at days 28 and 90 were significantly higher for meningitis than bacteraemia (p < 0.02).

In all age-groups, the highest CFP at 28 days were seen among persons with non-haematological malignancy, chronic liver disease, ARD and cardiac failure (Table 2). Of cases aged 18–64 years, who died on the day of culture or during the first week, the median ages were 50 and 48 years, respectively, and the most common underlying medical conditions were ARD, diabetes and immunodeficiency/rheumatic diseases in both groups. Of the fatal cases among persons aged 18–49 years and 50–64 years, 61 (49%) and 76 (59%), respectively, had an underlying condition which is considered a PPV23 indication. The CFP for patients aged 18–64 years with vaccine indication was 17% compared with 8% for patients without an indication (relative risk, 2.1; 95%CI, 1.6–2.6).

Hospital discharge data in connection with the IPD episode were available for 2132 (96%) of cases aged 18–64 years. We constructed a piecewise exponential hazard regression model to evaluate the risk of dying associated with various underlying medical conditions in this group. In this analysis, 848 (40%) patients had at least one underlying condition for which PPV23 is recommended and 1254 (59%) did not have a PPV23 indication. Of all patients, 477 (22%) had other, non-PPV23 indication discharge diagnosis codes (ICD-9 and ICD-10) given during the IPD episode or diagnosis codes given during previous hospitalisations within one year of the episode. The remaining 807 (38%) cases had neither other diagnoses nor previous hospitalisations and were considered the healthy reference group. The underlying medical conditions for which PPV23 is recommended, other diagnoses for hospitalisations, sex, age (two categories, 18–49 years and 50–64 years) and type of IPD (bacteraemia or meningitis) were included in the model (Table 4). The highest hazard ratios (HR) were seen for ARD, HIV infection and non-haematological malignancy. The HR for male sex, age 50–64 years, meningitis, ARD and haematological malignancy were time-dependent. In ARD the HR was highest during the first week, and it was only significant in meningitis from 8 to 28 days and in haematological malignancy from 29 to 90 days. In the model the HR are multiplicative, e.g. for a male aged 50–64 years with ARD and meningitis the risk of dying within 28 days was 25 times higher compared to 'previously healthy' group (1.6 × 1.3 × 3.2 × 3.8 = 25.3).

**Table 4:** Piecewise exponential hazard regression model for factors associated with death in cases aged 18–64 years with invasive pneumococcal infection

	Death at 0–7 days			Death at 8–28 days			Death at 29–90 days		
			
Factor	Hazard ratio	95%CI^a^	P-value	Hazard ratio	95%CI^a^	P-value	Hazard ratio	95%CI^a^	P-value
Male sex	0.9	0.6–1.2	0.5	1.6	1.3–2.0	< 0.001	1.6	1.3–2.0	< 0.001
Age > 50 years	1.3	1.1–1.7	< 0.05	1.3	1.1–1.7	< 0.05	2.8	2.2–3.5	< 0.001
Meningitis	1.2	0.7–2.3	0.7	3.2	2.0–5.0	< 0.001	0.7	0.4–1.2	0.3
Alcohol-related diseases	5.6	3.8–8.1	< 0.001	3.8	2.9–4.9	< 0.001	3.8	2.9–4.9	< 0.001
Cardiac failure	2.6	1.8–3.8	< 0.001	2.6	1.8–3.8	< 0.001	2.6	1.8–3.8	< 0.001
Chronic renal failure	1.6	0.5–5.2	0.4	1.6	0.5–5.2	0.4	1.6	0.5–5.2	0.4
Chronic liver disease	4.4	2.3–8.6	< 0.001	4.4	2.3–8.6	< 0.001	4.4	2.3–8.6	< 0.001
HIV infection	4.8	2.1–10.9	< 0.001	4.8	2.1–10.9	< 0.001	4.8	2.1–10.9	< 0.001
Haematological malignancy^b^	0.9	0.2–3.5	0.6	0.9	0.2–3.5	0.6	6.9	4.1–11.5	< 0.001
Non-haematological malignancy^b^	4.7	3.3–6.7	< 0.001	4.7	3.3–6.7	< 0.001	4.7	3.3–6.7	< 0.001
Diabetes mellitus	1.4	0.9–2.0	0.13	1.4	0.9–2.0	0.13	1.4	0.9–2.0	0.13
Chronic pulmonary disease	1.0	0.6–1.6	1.0	1.0	0.6–1.6	1.0	1.0	0.6–1.6	1.0
Organ/bone marrow transplantation	0.9	0.4–1.8	0.7	0.9	0.4–1.8	0.7	0.9	0.4–1.8	0.7
Immunodeficiency/rheumatic diseases	1.7	1.2–2.2	< 0.05	1.7	1.2–2.2	< 0.05	1.7	1.2–2.2	< 0.05
Other^c ^medical underlying conditions	1.7	1.3–2.2	< 0.001	1.7	1.3–2.2	< 0.001	1.7	1.3–2.2	< 0.001
Healthy	Ref.^d^			Ref.			Ref.		

^a^CI, confidence interval; ^b ^< 1 year since cancer diagnosis; ^c^non-PPV23 indication; ^d^Ref., reference

## Discussion

Data from our national, population-based study indicate that about half of all cases and deaths due to IPD occurred among working-age adults and that only 36% of the cases in this age-group had any of the underlying conditions for which PPV23 is currently recommended. To strengthen the efforts to reduce the high burden of IPD among non-elderly adults, policymakers should consider new prevention strategies to supplement the current recommendations for use of PPV23 to reach the almost two-thirds of cases without a vaccine indication.

By linking surveillance data to national vital statistics we were able to estimate all-cause mortality up to 3 months following an episode of IPD. Although the CFP was 9% during the first week, mortality at one month in persons with various underlying conditions ranged from 5% to 30% and increased in most groups up to 3 months after the first positive culture, probably reflecting both the severity of the underlying illness and the effects of long term sequelae [15]. The overall mortality was highest for non-haematological malignancy, chronic liver disease, alcohol-related diseases, cardiac failure and HIV infection. Our findings are consistent with two previous population-based studies on IPD and pneumococcal bacteraemic pneumonia in which mortality among persons with underlying medical conditions ranged from 3% to 13% [2] and 6% to 34% [16], respectively. In both studies the highest mortality was observed among persons with cirrhosis and alcohol abuse, coronary artery disease/congestive heart failure and non-haematological malignancies.

Our survival model for predictors of poor outcome in non-elderly adults indicated that the conditions with highest risk of death were alcohol-related diseases and non-haematological malignancies. The effects of some patient and disease characteristics such as meningitis as the clinical syndrome, age 50–64 years, male sex, ARD and haematological malignancies, seemed time-dependent in predicting death. The hazard ratios for these conditions were either significant only at some time-point in the model or they changed over time. Of all IPD cases, about 5% died during the day of admission and ARD was the most common underlying condition in this group of patients. The hazard ratios for ARD were higher at 0–7 days than at later time points, possibly reflecting delays in hospital admissions and treatment in this patient group.

In our study as much as 64% of IPD cases among working-age adults did not have any of the underlying conditions for which PPV23 is currently recommended. This proportion is substantially higher than previously reported from the U.S. (41%) [1,17] but there are few comparable data available from European countries. Alcohol-related diseases were the most frequent (11%) underlying condition in the working-age group as they have been in some other previous studies [18]. The incidence of IPD among persons with ARD in our study, however, was lower (21.9) compared with estimates ranging from 62 to 483.4 per 100,000 population in previous population-based studies [2,19,20]. However, because of lower base-line incidence in Finland, difficulties in defining the population at risk for ARD as well as evaluating the accuracy and representativeness of the denominator data used in these studies, the interpretation of the observed differences is complex. Our estimated denominator for ARD was an extrapolation based on the 12-month prevalence of persons with alcohol use disorders from a representative sample of Finnish adult (≥ 30 years) population [21].

Among patients with various immunocompromising conditions, the rate of IPD varied from 33.4 to 547.2 per 100,000 and was highest in those with haematological malignancy. In immunocompetent patients (persons with diabetes mellitus, chronic pulmonary disease and cardiac failure) there was less variation in rates (range, 12.0–47.1 per 100,000). Previous population-based studies reported higher rates for solid cancer (216.1 to 300.4 per 100,000), chronic pulmonary disease (62.9 to 503 per 100,000) and HIV (422.9 to 2031.4 per 100,000) [2,19,20], likely because of differences in population composition, databases, definitions, sources of denominator data and accuracy and completeness of identifying diagnoses of underlying conditions in IPD patients. Previous studies have included malignancies at any time point [2,20], whereas we restricted those diagnosed less than five or one year, respectively, before the IPD episode. For chronic pulmonary diseases, some studies incorporated only COPD and emphysema cases [19,20], but we also included asthma. The relatively low rate of IPD among persons infected with HIV in Finland may reflect good access to antiretroviral therapy, early antibiotic treatment without blood cultures and use of prophylactic antibiotics among those with low CD4^+ ^T cell count.

Although our estimates from national laboratory-based surveillance are representative of the entire population of Finland, the observed IPD incidence was low compared with reports from some other European countries [22-24], and the United States [1]. Our previous report from Finland found that the overall average annual incidence of IPD increased by 35.1% during a 8-year study period and increased in all adult age groups [25]. In that study temporal increase and higher regional IPD rates were significantly associated with higher blood culturing rates suggesting that the true incidence of IPD may be higher. Furthermore, although IPD is the most severe manifestation of pneumococcal infections, it represents only a small proportion of the overall burden of pneumococcal disease.

Of the national registries we used to define the co-morbidities for IPD cases and acquire population-based denominators, the Finnish Cancer Registry has almost 100% coverage [26,27], and the comprehensiveness of hospital discharge data has been validated previously [28-31]. However, our study also has several limitations. First, due to the registry-based study design, our analysis of the clinical outcome lacked chart review data to assess the effect of severity of illness indicators on IPD-related mortality. Information on some underlying conditions may also have been missed. Second, the denominator data for persons with co-morbidities were only available in aggregated form and did not allow estimating age-specific rates in various groups of patients with co-morbidities. Third, it is well known that ICD-coding in hospital discharge data may be incomplete and could be subject to misclassification. For this reason, we used hospital discharge data only to identify underlying conditions (ARD, chronic liver diseases, diseases of the spleen and CSF leakage) for which data were not available in the two other registries where standardised criteria and definitions are used. The standardised reimbursement criteria for underlying conditions in the National Social Insurance Institution's database may have excluded mild cases of certain underlying conditions such as COPD and asthma and diabetes mellitus type 2. Fourth, we did not have information on receipt of pneumococcal polysaccharide vaccination and cigarette smoking habits of patients as some of the associations we found with higher risk of IPD (e.g. alcohol-related diseases and COPD) may be confounded by smoking. About half of invasive pneumococcal disease in immunocompetent non-elderly adults has been previously attributed to cigarette smoking [32].

In Finland, the coverage of PPV23 among the elderly and high risk groups is about 3% and would not be expected to impact our results. Despite of the existing vaccine recommendation, PPV23 is not included in the government-funded national vaccination program, and the expense is covered by the treating clinical unit or the individual. Two clinical trials have been conducted in Finland to assess the efficacy of PPV23 against pneumonia [33,34]. The conflicting results from these trials regarding the efficacy in the aged of PPV23 against mainly serologically diagnosed pneumococcal pneumonia, or pneumonia in general probably have also had a major influence on the vaccination coverage.

The patient groups with highest rates of IPD (e.g. haematological malignancy, organ and bone marrow transplantation, HIV infection) were different from those at highest risk of death (e.g. ARD, non-haematological malignancy and cardiac failure). The almost two-thirds of working-age cases without PPV23 indication, as well as those with alcohol-related diseases may be difficult to reach with public health interventions and acceptability of vaccination may be low among healthy persons. One proposed strategy includes lowering the recommended age for universal PPV23 vaccination to include all persons aged 50 years and older which might result in moderately increased number of IPD cases prevented compared with the current high risk indications [35,36]. However, given the increasing risk of IPD and mortality with age and the unknown duration of protection after primary immunisation, the optimal timing and frequency of revaccination with PPV23 will need to be determined before this strategy can be implemented. Currently, there are no data available on the clinical effectiveness of revaccination and serologic studies suggest that antibody responses may by lower after revaccination that after primary vaccination [37-39].

Routine childhood immunisation with PCV7 has not yet been introduced in Finland. However, increasing evidence has been accumulating about the substantial indirect effects of childhood PCV7 immunisation in reducing rates of adult pneumococcal disease in the U.S. and elsewhere [40,41], although early reports from some European countries have had inconsistent results [42-45]. The serotypes included in PCV7 cause approximately 50% of IPD in Finnish adults, a proportion similar to the U.S. before PCV7 introduction [25]. Therefore, introducing routine childhood immunisation in Finland would provide an opportunity to substantially reduce the disease burden among the difficult-to-reach groups of working-age adults without PPV23 indications [11].

## Conclusion

In addition to young children and elderly persons, the burden of invasive pneumococcal infections is also substantial among working-age persons without high risk conditions. In the general population of non-elderly adults, two-thirds of invasive infections and one half of fatal cases occur in persons without a recognised PPV23 indication. Policymakers should therefore consider additional prevention strategies to reduce the burden of pneumococcal disease in the overall population.

## Abbreviations

ACIP: Advisory Committee on Immunization Practices; ARD: Alcohol-Related Diseases; CFP: Case-Fatality Proportion; CI: Confidence Interval; COPD: Chronic Obstructive Pulmonary Disease; CSF: Cerebrospinal Fluid; HCD: Health Care District; HILMO: National Hospital Discharge Register; HR: Hazard Ratio; ICD: International Classification of Diseases; IPD: Invasive Pneumococcal Disease; KELA: National Social Insurance Institution; NIDR: National Infectious Disease Register; PCV7: 7-valent Pneumococcal Conjugate Vaccine; PPV23: 23-valent Pneumococcal Polysaccharide Vaccine.

## Competing interests

PK has received lecturing fees from Wyeth Finland. The other authors have no competing interests.

## Authors' contributions

Study concept and design: OL and JPN. Acquisition of data: PK. Analysis and interpretation of data: PK, OL, PR, JO and JPN. Drafting of the manuscript: PK, OL, PR and JPN. Critical revision of the manuscript for important intellectual content: PK, OL, PR, JO and JPN. Statistical analysis: PK and JO. Study supervision: OL and JPN. All authors have contributed to the manuscript and approved the final version.

## Pre-publication history

The pre-publication history for this paper can be accessed here:

http://www.biomedcentral.com/1471-2334/8/96/prepub

## Supplementary Material

Additional file 1 Recommendation for the use of 23-valent pneumococcal polysaccharide vaccine in Finland. Click here for file

Additional file 2 ICD-9 and ICD-10 codes used in defining underlying conditions for data in the National Hospital Discharge database. Click here for file
