# Supplementary material for: Invasive pneumococcal infections among persons with and without underlying medical conditions: Implications for prevention strategies
Source: BMC Infect Dis. 2008 Jul 22;8:96. doi: 10.1186/1471-2334-8-96 (PMC2507715; doi:10.1186/1471-2334-8-96)
Supplement: Additional file 1 — Recommendation for the use of 23-valent pneumococcal polysaccharide vaccine in Finland. [file 1471-2334-8-96-S1.doc]

Additional file 1

Recommendations for the use of 23-valent pneumococcal polysaccharide vaccine in Finland

**Immunocompetent persons aged ≥5 years**

- Persons with cardiac failure, chronic pulmonary disease (chronic obstructive pulmonary disease, emphysema, but not asthma), diabetes mellitus, liver failure, renal failure, cerebrospinal fluid leaks, cochlear implants and alcoholism
- Persons aged ≥65 years
- Persons residing in long-term care facilities

**Immunocompromised persons aged ≥5 years**

- Persons with functional or anatomic asplenia, HIV infection, lymphoma, multiple myeloma, nephrotic syndrome, congenital or acquired immunodeficiency (not agammaglobulinemia)
- Persons who received an organ or bone-marrow transplant
- Persons receiving ongoing immunosuppressive chemotherapy (including systemic corticosteroids)
