# Supplementary material for: Invasive pneumococcal infections among persons with and without underlying medical conditions: Implications for prevention strategies
Source: BMC Infect Dis. 2008 Jul 22;8:96. doi: 10.1186/1471-2334-8-96 (PMC2507715; doi:10.1186/1471-2334-8-96)
Supplement: Additional file 2 — ICD-9 and ICD-10 codes used in defining underlying conditions for data in the National Hospital Discharge database. [file 1471-2334-8-96-S2.doc]

Additional file 2. ICD-9 and ICD-10 codes used in defining underlying conditions for data in the National Hospital Discharge database.

| Underlying condition | ICD-9 codes | ICD-10 codes |
| --- | --- | --- |
| Alcohol-related diseases (Morbus Wernicke, mental, behavioural and neurological disorders due to use of alcohol, hepatic and pancreatic disorders due to use of alcohol, toxic effects of alcohol, maternal and foetal care due alcohol abuse, rehabilitation and counseling alcohol abuse) | 2651X; 2910A, 2911A, 2913A,  2918A, 2948X, 3039X, 3050A;  3318X; 3451A; 3575A; 3594A; 5710A, 5711A, 5712A, 5713X; 5770D, 5771B, 5771C, 5771D; 9800A, 9801A, 9802A, 9803A, 9808X, 9809X; 6554A; 7607A, E850A; V654A | E51.2; F10; G31.2; G40.51; G62.1; G72.1; K70; K86; T51;  O35.4; P04.3; X45; Y91; Z50.2; Z71.4 |
| Chronic liver diseases (hepatic failure, cirrhosis of liver and biliary ducts, portal hypertension, hepatorenal syndrome and unspecified liver disease) | 5709A, 5719X, 5739X; 5715A, 5716A, 5716X, 5719X; 5724A; 5739X | K72; K74; K76.7; K76.9 |
| Diseases of spleen | 2894A, 2895A | D73 |
| Cerebrospinal fluid leak | 3498A | G96.0 |
